# Impact of plasma Epstein–Barr virus DNA in posttreatment nasopharyngeal carcinoma patients after SARS-CoV-2 infection

**DOI:** 10.1186/s13027-024-00570-x

**Published:** 2024-03-14

**Authors:** Cheng Lin, Meifang Li, Yingying Lin, Yu Zhang, Hanchuan Xu, Bijuan Chen, Xia Yan, Yun Xu

**Affiliations:** 1 Department of Radiation Oncology, Clinical Oncology School of Fujian Medical University, Fujian Cancer Hospital, Fuzhou, Fujian Province China; 2 Interdisciplinary College of Medicine and Engineering, Fuzhou University, Fuzhou, Fujian China; 3 Department of Medical Oncology, Clinical Oncology School of Fujian Medical University, Fujian Cancer Hospital, Fuzhou, Fujian Province China

**Keywords:** Nasopharyngeal carcinoma, EBV DNA, COVID-19, Omicron, Relapse

## Abstract

**Background:**

Nasopharyngeal carcinoma (NPC) is prevalent in southern China. EBV DNA is the most useful biomarker in NPC. However, the value of EBV DNA in posttreatment NPC patients infected with severe acute respiratory syndrome coronavirus 2 (SARS-CoV-2) remains unclear.

**Methods:**

Sixty-four eligible NPC patients were enrolled between December 2022 and February 2023. Patients who met the following criteria were included: had non-metastatic NPC, completed radical treatment, were first firstly infected with SARS-CoV-2 and their EBV DNA changed from undetectable to detectable.

**Results:**

At the end of follow-up, 81.25% (52/64) of patients were confirmed not to relapse with undetectable EBV DNA (no-relapse). In addition, 18.75% (12/64) of patients experienced relapse with consistent detection of EBV DNA (yes-relapse). For all 64 patients, the average time from diagnosis of coronavirus disease 2019 (COVID-19) to detection of detectable EBV DNA was 35.41 days (2 to 139 days). For 52 no-relapse patients, the average time from EBV DNA changing from detectable to undetectable was 63.12 days (6 to 147 days). The levels of EBV DNA were greater in yes-relapse patients than that in no-relapse patients, and the average of EBV DNA levels were 1216 copies/ml and 53.18 copies/ml, respectively. Using 62.3 copies/mL as the threshold, the area under the curve for EBV DNA was 0.88 for distinguishing yes-relapse patients from no-relapse patients. The sensitivity and specificity were 81.97% (95% CI 0.71–0.95) and 86.67% (95% CI 0.70–0.95), respectively.

**Conclusion:**

For NPC patients infected with SARS-CoV-2, EBV DNA alone is insufficient for monitoring relapse after radical therapy. Long-term follow-up and underlying mechanistic investigations of EBV DNA changes are urgently needed.

## Background

Coronavirus disease 2019, commonly known as COVID-19, is a highly contagious respiratory illness caused by severe acute respiratory syndrome coronavirus 2 (SARS-CoV-2) [1]. COVID-19 was first reported in December 2019 and has severely affected the global health system [2]. Symptoms of COVID-19 include fever, cough, shortness of breath, fatigue, muscle or body aches, sore throat, congestion, headache, gastrointestinal issues and so on [3–5]. Some individuals may experience asymptomatic or mild symptoms, while others may develop severe or life-threatening complications.

Notably, the effects of COVID-19 on cancer patients have been substantial, including both direct consequences due to SARS-CoV-2 infection and indirect consequences resulting from disruptions in cancer care caused by the pandemic. The COVID-19 pandemic has caused significant disruptions in the diagnosis, screening, initiation of treatment, modifications, evaluation, and supervision of cancer patients [6–9]. Cancer patients infected with SARS-CoV-2, including head and neck cancer patients have been found to have a greater risk of severe illness and mortality than the general population, [10]. Therefore, oncologists must address the above challenges and provide constructive suggestions to address possible future pandemics. However, the impacts of COVID-19 on cancer patients can vary depending on factors such as cancer type, stage, and individual patient characteristics [11, 12].

Nasopharyngeal carcinoma (NPC) is the most common cancer of the head and neck in China, especially in the endemic areas of Fujian, Guangdong, Guangxi, and Hunan provinces [13]. NPC is closely associated with Epstein–Barr virus (EBV) infection [14]. Currently, plasma EBV DNA is considered the most sensitive and specific biomarker for the management of NPC, as it can be used for screening, diagnosis, therapeutic response evaluation, prognostic prediction, and monitoring [15]. Notably, the COVID-19 pandemic has resulted in significant health care implications for NPC patients, including a reduction in newly diagnosed NPC patients, delayed chemoradiotherapy and adverse clinical outcomes. When the Omicron strain of COVID-19 was prominent in Fuzhou [16], we found that in some NPC patients who completed comprehensive treatment, the levels of EBV DNA suddenly changed from undetectable to detectable when patients were infected with the Omicron strain (B.1.1.529) of SARS-CoV-2. The EBV DNA may again return to undetectable after a period of follow-up, while few patients suffer from recurrence or metastasis with progressive elevation of EBV DNA. Thus, we wondered whether plasma EBV DNA measurement was reliable when patients were infected with SARS-CoV-2. Therefore, our study aimed to evaluate the utilization and effectiveness of EBV DNA in the follow-up of NPC patients diagnosed with the Omicron strain of COVID-19.

## Materials and methods

### Patients

A total of 64 NPC patients were enrolled from Fujian Cancer Hospital between December 2022 and February 2023, when the Omicron strain of SARS-CoV-2 was prevalent in our hospital. The inclusion criteria were as follows: (I) patients without distant metastasis, (II) patients who were first infected with the Omicron variant (B.1.1.529) of SARS-CoV-2, (III) patients who had finished complete treatment and achieved a complete response, (IV) EBV DNA that returned to 0 after definitive treatment, (V) EBV DNA that changed from undetectable to detectable when the patient was diagnosed with the Omicron variant (B.1.1.529) of SARS-CoV-2, and (VI) Relapse was confirmed by histological examination. The exclusion criteria were as follows: (I) patients who demonstrated recurrence and or metastasis before being diagnosed with COVID-19, (II) patients whose EBV DNA level did not return to 0 after definitive treatment, (III) patients who were receiving treatment during the screening period, or (IV) patients who were lost to follow-up. Notably, tests for SARS-CoV-2 infection were not routinely conducted for posttreatment NPC patients. Thus, we focused only on and dynamically tracked the NPC patients with SARS-CoV-2 infection and detectable EBV DNA, since the detectable EBV DNA might indicate tumor progression and cause a large psychological burden for these patients after SARS-CoV-2 infection. In addition, to eliminate the interference of confounding factors of EBV DNA, we deliberately excluded those who did not have a relapse, but whose EBV DNA was at a detectable level. Patients with NPC were classified according to the AJCC/UICC 8th Edition Staging System. This study was approved by the Ethical Review Committee of Fujian Cancer Hospital (approval no. K2022-142–01). Written informed consent was obtained from all participants following a detailed description of the purpose of the study.

### Measurement of EBV DNA and the Omicron strain of SARS-CoV-2

The concentrations of plasma EBV DNA were all measured at our hospital using an EBV nucleic acid amplification fluorescence quantitative PCR detection Kit as previously described [17]. The presence of the Omicron strain of SARS-CoV-2 was tested with a nucleic acid detection kit using fluorescence PCR method at our hospital and other accredited hospitals and institutions [18].

### Treatment

Stage I, II, and III-IVA NPC patients received radiation therapy alone, concurrent chemoradiotherapy, and platinum-based induction chemotherapy combined with radiation therapy, respectively. The platinum-based chemotherapy regimen typically includes gemcitabine or paclitaxel combined with cisplatin or nedaplatin. Concurrent chemotherapy consisted of cisplatin or nedaplatin.

### Follow‐up

Surveillance was conducted after the completion of definitive therapy. Patients were evaluated every 3 months for 2 years, then every 6 months for 3 years, and then every 12 months. Each follow-up consisted of an exhaustive assessment, fiberoptic endoscopy, fundamental serum chemistry analysis, thoracic computed tomography (CT) scan or X-ray, bone emission computed tomography (ECT), and ultrasound or CT imaging of the abdomen. Magnetic resonance imaging (MRI) of the cranial and cervical regions was subsequently conducted after the completion of intensity-modulated radiation therapy (IMRT), at 3–6 month intervals during the initial 5 years, and subsequently on an annual basis thereafter. The use of EBV DNA was recommended every month until EBV DNA returned to being undetectable. If NPC recurrence was considered after examination, a biopsy and pathology of the recurrent lesion were performed. Progression-free survival (PFS) was measured from the date of diagnosis to the time of disease progression or death from any cause.

### Statistical analysis

All the statistical analyses were performed using SPSS version 27.0 and GraphPad Prism 9. Differences in EBV DNA expression between yes-relapse and no-relapse patients were compared using t-tests. The relationship between EBV DNA expression and patient characteristics was analysed using the χ2 test. The cut-off value of plasma EBV DNA in yes-relapse versus no-relapse patients was derived by receiver operating characteristic (ROC) curves with Youden’s index. Multivariate analyses were conducted with a Cox proportional hazards model. All *P*-values were 2-sided.

## Results

### Patient characteristics

**Fig. 1:**
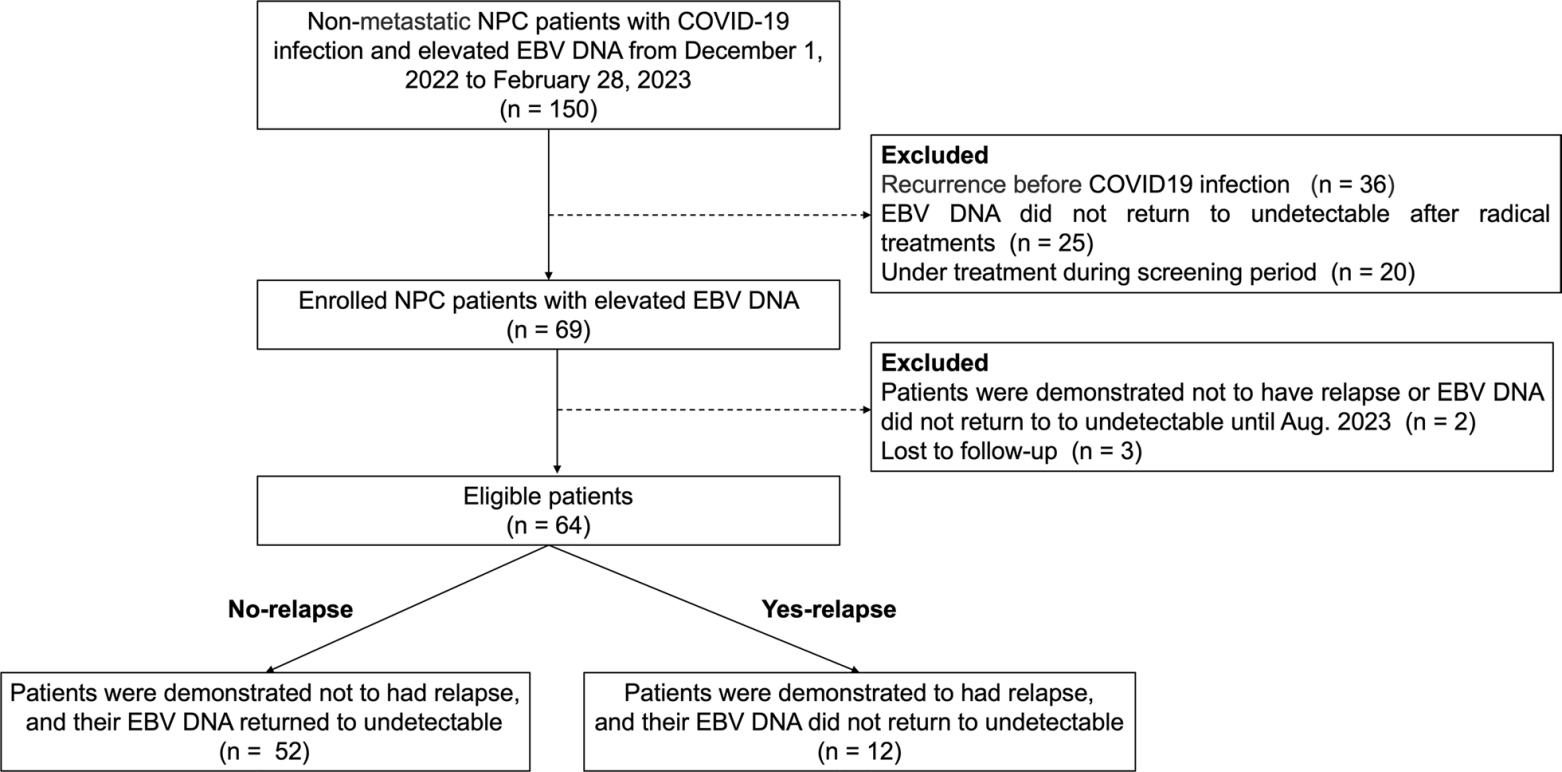
Flowchart of the patient selection process

Sixty-four patients with non-metastatic NPC with detectable EBV DNA after infection with SARS-CoV-2 between December 2022 and February 2023, were included in our study (Fig. 1). The median age was 52.11 years, and 84.4% (54/64) of patients had COVID-19 symptoms. All 64 patients had detectable baseline plasma EBV DNA levels. Notably, 81.25% (52/64) of patients whose EBV DNA was undetectable recovered did not relapse until the end of follow-up (no-relapse). However, EBV DNA was consistently detectable in 12 patients, and those patients were demonstrated to have recurrence or metastasis during follow-up (yes-relapse). Among the 12 yes-relapse patients, 1 patient experienced local and regional recurrence. In addition, 1, 5, and 5 patients suffered from local, regional, and distant metastasis, respectively.

The characteristics of the patients who did or did not experience relapse are listed in Table 1. No significant differences were found between relapse and age, sex, T stage, N stage, or TNM stage, or between patients with or without symptoms of COVID-19 or fever. Multivariate analysis revealed that the above characteristics were not associated with PFS (Table 2).

**Table 1:** Characteristics of 64 non-metastatic posttreatment nasopharyngeal carcinoma patients diagnosed with COVID-19

Characteristics	Relapse		*p*
	Yes n (%)	No n (%)	
Total	12 (18.75)	52 (81.25%)	
Age(y)			0.200
≤ 50	4 (33.3)	28 (53.8)	
> 50	8 (66.7)	24 (46.2)	
Sex			0.739
Male	9 (75.0)	35 (67.3)	
Female	3 (25.0)	17 (32.7)	
T stage			0.510
T1–2	3 (25.0)	21 (26.0)	
T3–4	9 (75.0)	31 (74.0)	
N stage			0.907
N0–1	5 (41.7)	25 (48.1)	
N2–3	7 (58.3)	27 (51.9)	
TNM stage			0.274
1–2	1 (8.3)	13 (25.0)	
3–4	11 (91.7)	39 (75.0)	
Symptoms of COVID-19			0.186
No	0 (0.0)	10 (19.2)	
Yes	12 (100.0)	42 (80.8)	
Fever			0.181
No	3 (25.0)	24 (46.2)	
Yes	9 (75.0)	28 (53.8)	

COVID19, Coronavirus disease 2019; EBV DNA, Plasma Epstein–Barr virus DNA

**Table 2:** Multivariate analysis for PFS in 64 nasopharyngeal carcinoma patients with SARS-CoV-2 infection

Characteristics	PFS	
	HR (95% CI)	*P*
Age (< 50 vs. ≥ 50 years)	2.87 (0.70–11.79)	0.144
Sex (male vs. female)	0.48 (0.10–2.27)	0.355
T stage (T1–2 vs. T3–4)	1.04 (0.19–5.68)	0.969
N stage (N0–1 vs. N2–3)	0.59 (0.14–2.44)	0.467
TNM stage (1–2 vs. 3–4)	3.23 (0.19–54.70)	0.418
Symptoms of COVID-19 (No vs. Yes)	–	0.979
Fever (No vs. Yes)	0.80 (0.20–3.31)	0.762

### Change characteristics of EBV DNA

For all 64 patients, the time from diagnosis of COVID-19 to detection of detectable EBV DNA ranged from 2 to 139 days, with an average of 35.41 days. For 52 patients who did not relapse, the time from EBV DNA changing from detectable to undetectable ranged from 6 to 147 days, with an average of 63.12 days. For 12 yes-relapse patients, the time from detectable EBV DNA to diagnosis of relapse was 0—203 days, with an average of 101 days.

**Fig. 2:**
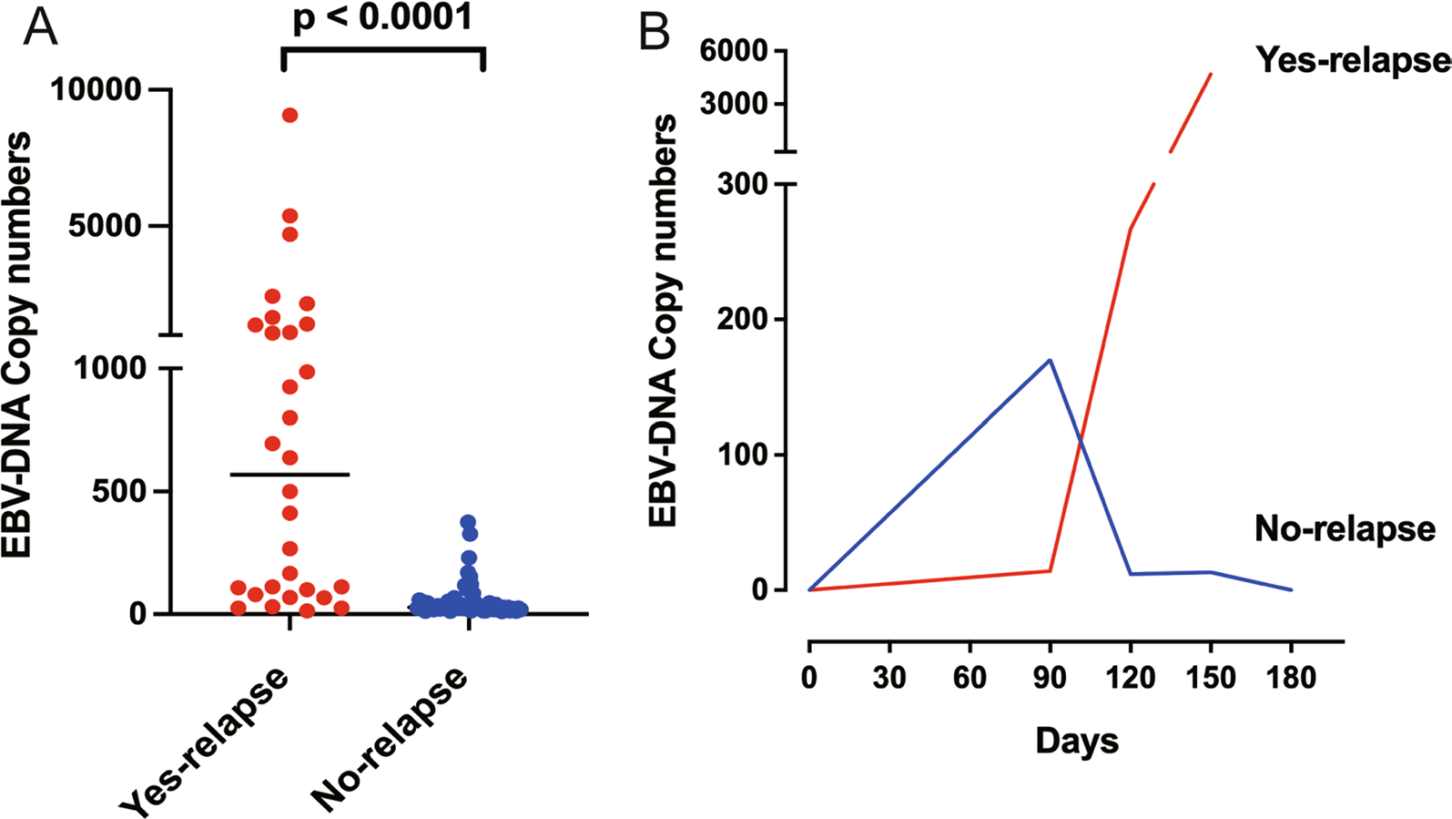
EBV DNA changes in NPC patients infected with SARS-CoV-2. **A** Changes in EBV-DNA concentrations in patients who did not relapse with undetectable EBV DNA (no-relapse) and in patients who experienced relapse with consistently detectable EBV DNA (yes-relapse). **B** Typical case of EBV DNA changes in one yes-relapse patient and one no-relapse patient who underwent timely follow-up and examination

To further address the changes in EBV DNA, EBV DNA data detected at all time points during the follow-up were analysed. Compared to those in no-relapse patients, the levels of EBV DNA were greater in yes-relapse patients (Fig. 2A). The concentrations of EBV DNA ranged from 14.2 to 9070.0 copies/mL in yes-relapse patients, with an average of 1216 copies/ml. In contrast, no-relapse patients had concentrations of EBV DNA ranging from 11.4 to 375.0 copies/mL, with an average of 53.18 copies/ml.

Typical cases of one yes-relapse patient and one no-relapse patient who underwent timely follow-up and examination are listed in Fig. 2B. There was a progressive increase in EBV DNA in yes-relapse patients. In contrast, no-relapse patients had transiently positive EBV DNA, which became undetectable after long-term follow-up.

### Association of the levels of EBV DNA with relapse

**Fig. 3:**
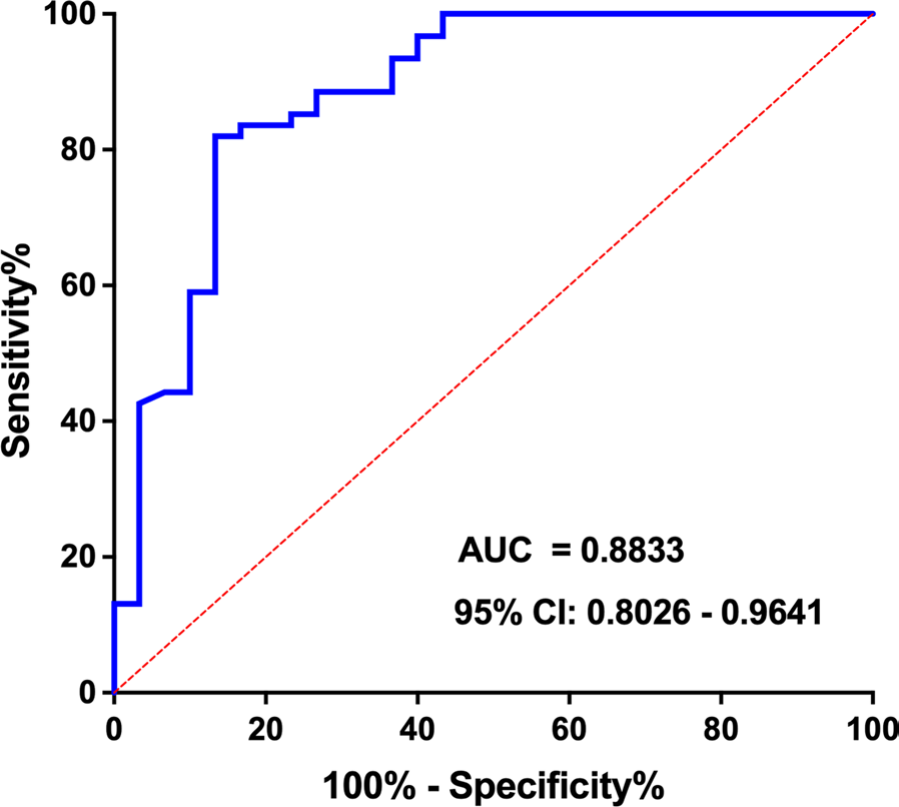
Receiver operating characteristic (ROC) curve analysis of EBV DNA for the diagnosis of relapse

To distinguish no-relapse patients from yes-relapse patients, we further evaluated the cut-off value of EBV DNA. Through the ROC curve, we found that EBV DNA had a predictive value of 88% (95% CI 0.80–0.96) using a cut-off value of 62.3 copies/mL (Fig. 3). The sensitivity and specificity for identifying yes-relapse patients were 81.97% (95% CI 0.71–0.95) and 86.67% (95% CI 0.70–0.95), respectively.

## Discussion

EBV infection is an aetiologic factor in the carcinogenesis of NPC [19]. Studies have shown that the presence of EBV DNA is a promising indicator for posttreatment surveillance [15, 20]. Currently, EBV DNA monitoring is recommended by the National Comprehensive Cancer Network (NCCN), with Category 2B of Evidence and Consensus in NPC [21]. However, whether plasma EBV DNA measurements are reliable, and how to arrange EBV DNA tests during the COVID-19 pandemic remain unknown.

In this article, we found that plasma EBV DNA was a valuable tool for predicting recurrence or metastasis using an appropriate cut-off value after radical treatment in NPC patients infected with the Omicron strain of SARS-CoV-2. However, EBV DNA alone was not a reliable factor for the confirmation of relapse since some patients had false-negative results [22–24]. Studies have reported that 51%-67% of NPC patients with local or locoregional failure have elevated EBV DNA, and 86%-96% of patients with distant metastases have detectable EBV DNA [20]. Therefore, during the COVID-19 pandemic, the diagnosis of relapse should be made by combining EBV DNA with other tools, including CT, MRI, bone ECT, PET-CT, nasendoscopy, and biopsy. Our findings support the consensus that EBV DNA should not be the only surveillance test available, and it can not replace clinical consultations, nasendoscopy, biopsy, and other imaging or diagnostic tools for predicting the relapse of NPC after radical treatment [25]. Simple, reliable, and economical approaches are needed to improve the accuracy of EBV DNA monitoring. Notably, we speculated that the fluctuations in EBV DNA levels might be due to SARS-CoV-2 infection, since we rarely found so many patients with abnormal fluctuations in EBV DNA when no signs of cancer recurrence or metastasis existed in NPC patients before the COVID-19 epidemic. Therefore, explorations are needed to study the difference in EBV DNA between NPC patients with or without COVID-19 and to confirm whether there is any relationship between EBV DNA levels and SARS-CoV-2 infection. In addition, the underlying mechanisms are wanted to explain why EBV DNA fluctuates after SARS-CoV-2 infection, which could help to provide a valuable reference for the future for the COVID-19 epidemic.

In addition, the optimal timing and frequency of EBV DNA tests needed to determined. Chen et al. showed that 63.8% of patients with detectable cell-free EBV DNA developed recurrence. For EBV DNA-detectable patients who did not develop recurrence, 81.7% had transiently detectable EBV DNA that decreased undetectable levels after long-term follow-up [26]. Thus, dynamic and regular EBV DNA tests are recommended after the completion of radical treatment in non-disseminated NPC patients. In our study, the average time for EBV DNA to recover to undetectable levels was 63 days in no-relapse NPC patients whose EBV DNA level increased from undetectable to detectable during follow-up. Thus, we suggest every 2-month interval test for EBV DNA for patients infected with the Omicron strain of SARS-CoV-2. However, for patients whose EBV DNA is always detectable, whose level is higher than the cut-off value (62.3 copies/mL) or whose level is persistently elevated, we should be aware of the potential for recurrence or metastasis. For patients whose EBV DNA was transiently elevated, transient lytic and latent cycles of EBV could not be completely ruled out. Despite all these possibilities, closer monitoring of EBV DNA was suggested for NPC patients with abnormal EBV DNA levels. In addition, other specific examinations, such as the COVID-19 pandemic, are still wanted even if a prolonged interval is allowed in severely resource-constrained circumstances. Imaging and histological tools provided a clear and integrated representation of the appearance and properties of the tumor. Therefore, imaging and endoscopic assessments are essential for the surveillance of NPC patients with increased or progressively increasing levels of EBV DNA or symptoms highly suggestive of NPC recurrence and metastasis. Further validation is needed in prospective and multicentre clinical trials to determine the frequency of EBV DNA monitoring.

To our knowledge, our study is the first to explore the utilization of EBV DNA in patients with non-metastatic NPC after radical treatment during the COVID-19 pandemic. Our study had several limitations. First, our study was conducted in a high-incidence area of NPC in the Asian population, which may not be applicable to non-endemic areas or other ethnic groups. Second, this article was a retrospective study in a single centre. Third, since SARS-CoV-2 is highly variable, it is unclear whether our findings could be applied to other variants of SARS-CoV-2 and second infections caused by COVID-19 [27, 28]. Fourth, the time of EBV DNA detection is irregular due to resource limitations during the COVID-19 pandemic and personal factors, and the follow-up time is relatively short. However, further studies are needed to explore the utilization of EBV DNA during the COVID-19 pandemic.

## Conclusion

In conclusion, for non-metastatic posttreatment NPC patients infected with the Omicron strain of SARS-CoV-2, EBV DNA alone is insufficient for monitoring the relapse after radical therapy. Dynamic and regular EBV DNA tests combined with imaging, nasendoscopy, biopsy, and other diagnostic tools are strongly recommended.

## Data Availability

All data generated or analysed during this study are included in this article and supplementary files.

## Abbreviations

NPC: Nasopharyngeal carcinoma
COVID-19: Coronavirus disease 2019
SARS-CoV-2: Severe acute respiratory syndrome coronavirus 2
EBV: Epstein–Barr virus
IMRT: Intensity-modulated radiation therapy
CT: Computed tomography
ECT: Emission computed tomography
MRI: Magnetic resonance imaging
ROC: Receiver operating characteristic
NCCN: National Comprehensive Cancer Network
